# Successful surgical resection of a metastatic clear cell sarcoma in the heart: a case report

**DOI:** 10.1093/ehjcr/ytae174

**Published:** 2024-04-09

**Authors:** Ayano Osawa, Hiroto Utsunomiya, Shuichiro Takanashi, Shinya Takahashi, Yukiko Nakano

**Affiliations:** Department of Cardiovascular Medicine, Hiroshima University Hospital, Hiroshima, Japan; Department of Cardiovascular Medicine, Hiroshima University Graduate School of Biomedical and Health Sciences, 1-2-3 Kasumi, Minami-ku, Hiroshima 734-8551, Japan; Department of Cardiovascular Surgery, Kawasaki Saiwai Hospital, Kanagawa, Japan; Department of Surgery, Hiroshima University Graduate School of Biomedical and Health Sciences, Hiroshima, Japan; Department of Cardiovascular Medicine, Hiroshima University Graduate School of Biomedical and Health Sciences, 1-2-3 Kasumi, Minami-ku, Hiroshima 734-8551, Japan

**Keywords:** Cardiac tumour, Case report, Clear cell sarcoma, Echocardiography, PET-CT

## Abstract

**Background:**

Clear cell sarcoma (CCS) is a very rare disease and one with a very poor prognosis. Furthermore, its occurrence in the heart is very rare and past reports are scarce.

**Case summary:**

A 33-year-old man who had undergone left arm amputation due to CCS came to the hospital because a positron emission tomography computed tomography (PET-CT) four years post-amputation showed an accumulation in the heart. The PET-CT with glucose suppression treatment showed fluorodeoxyglucose accumulation in the myocardium between the middle of the anterolateral wall and the papillary muscle of the posterior lateral wall of the left ventricle (LV). Based on the course of the disease up to now, it was considered that the accumulation was most likely metastasis of CCS. Observation of the heart after a median sternotomy revealed a white tone, well-defined lesion in the middle of the anterolateral wall of LV. The tumour on the posterolateral side of LV was not exposed on the surface, but it was palpated and was still recognizable as a firm neoplastic lesion. Because the mass was identified as a sarcoma on intraoperative rapid pathology, we decide to perform a total resection. Both lesions were excised, and pathology revealed a diagnosis of CCS.

**Discussion:**

Clear cell sarcoma is a very rare disease that accounts for <1% of all soft tissue sarcomas, and its occurrence in the heart is even rarer. It requires a combination of many imaging modalities. To our knowledge, this is the first case of CCS in the heart treated with surgical resection.

**Learning points**

- To raise awareness that although clear cell sarcoma is still a reasonably well-known disease in the field of orthopaedics, it may also occur in the heart, although it has been reported less frequently and is rare.
- Clear cell sarcoma in the heart is difficult to diagnose: in this case, multi-modality imaging was helpful.

## Introduction

Clear cell sarcoma (CCS) is a rare soft tissue tumour that was first described by Enzinger^1^ and accounts for <1% of all soft tissue sarcomas. The prognosis for this disease is poor, a five-year survival rate of 47–54%, a 10-year survival rate of only 33–36%, and more than 50% of these patients develop metastases.^2–4^ Its occurrence in the heart is very rare, and there have been no previous reports of its resection. Appropriate diagnosis and pathologic confirmation of cardiac CCS are important.

## Summary figure

**Table:** 

0	Onset of left wrist joint pain.
3 months	Amputation of the left arm for clear cell sarcoma.
4 years, 8 months	PET-CT showed an accumulation in the heart.
4 years, 10 months	He was admitted to our hospital for examination.
4 years, 11 months	The tumour was removed by surgery.
5 years, 8 months	The last follow-up: PET-CT showed no recurrence or metastases.

## Case summary

**Figure 1:**
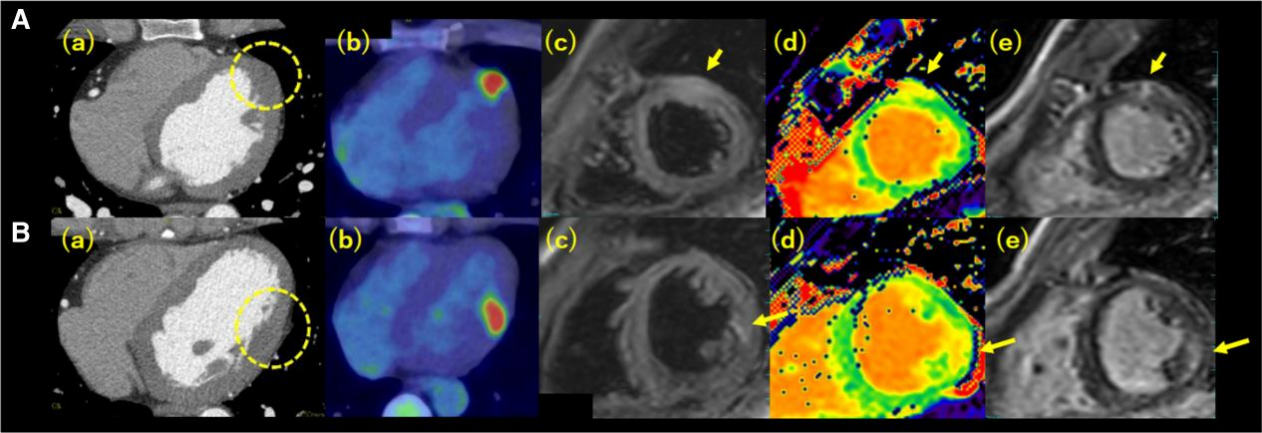
(*A*) A mass is in the middle of the left ventricular anterolateral wall (arrows and circle). (*B*) The other mass is located between the papillary muscles of the left ventricular posterolateral wall (arrows and circle). (*a*) Axial of computed tomography (CT) shows areas of pale low CT values. (*b*) Positron emission tomography CT (PET-CT) axial shows fluorodeoxyglucose (FDG) accumulation. (*c*) Delayed contrast sagittal of contrast magnetic resonance imaging (MRI) shows high signal. (*d*) T1 mapping sagittal of contrast MRI shows a different image than normal myocardium. (*e*) T2 emphasis sagittal of contrast MRI shows mildly high signal.

**Figure 2:**
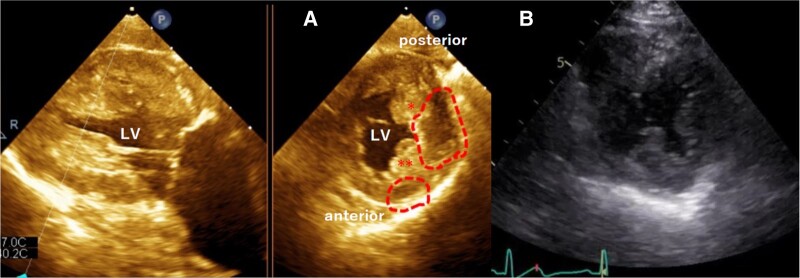
(*A*) Transoesophageal echocardiographic (TOE) trans gastric long-axis and short-axis images of the left ventricle shows hypoechoic areas in the anterolateral mid-wall of the left ventricle (circle) and in the posterior interventricular anterior (**) and posterior (*) papillary muscle (circle) that are different from normal myocardium. (*B*) The mass is indistinct on the short-axis image of the transthoracic echocardiogram (TTE).

A 33-year-old man was diagnosed with tendinitis due to pain in his left wrist joint, and he was referred to another hospital because his condition did not improve. He was diagnosed with giant cell sarcoma of the tendon sheath and underwent a limbal resection, which led to a diagnosis of CCS and amputation of the left arm. He was referred to our hospital for further examination because the positron emission tomography computed tomography (PET-CT) performed four years and five months later showed an accumulation in the heart. He had no symptoms, and his physical examination did not reveal any abnormalities. His grandfather had pancreatic cancer, but he had no history other than CCS. Blood tests showed a slightly elevated N-terminal pro-brain natriuretic peptide at 98 pg/mL (normal range: 1–55 pg/mL), but high-sensitivity troponin T was normal at 0.009 ng/mL (normal range: 0–0.014 ng/mL) and other laboratory parameters were also normal. Transthoracic echocardiography (TTE) showed that the left ventricle ejection fraction (LVEF) was maintained at 65% and no obvious mass-like structures were noted within the visible range. Contrast-enhanced computed tomography (CT) showed pale areas of low CT values within the myocardium in the middle of the anterolateral wall of the LV [*Figure 1A*(*a*)] and between the papillary muscles of the posterolateral wall of the LV [*Figure 1B*(*a*)]. Transoesophageal echocardiography (TOE) showed a hypoechoic region within the myocardium in the same area as in the CT, which appeared to be different from the normal myocardium. The border was indistinct, but there was no protrusion of the tumour into the cardiac cavity (*Figure 2A*). Positron emission tomography computed tomography performed after hypoglycaemic treatment showed intramyocardial fluorodeoxyglucose (FDG) accumulation in the middle of the anterolateral wall of the LV [*Figure 1A*(*b*)] and between the papillary muscles of the posterolateral wall of the LV [*Figure 1B*(*b*)], suggesting a high possibility of malignancy. Cardiac magnetic resonance imaging (MRI) showed a heterogeneous lesion in the middle to apex of the left ventricular anterior wall [*Figure 1A*(*c*, *d*, *e*)] and in the base to middle of the inferior lateral wall [*Figure 1B*(*c*, *d*, *e*)], with indistinct borders and internal heterogeneity, which was different from normal myocardium. Cardiac angiography showed no coronary artery abnormality and no nutrient vessels to the tumour. We suspected CCS metastasis based on the history and considered cardiac biopsy to confirm the diagnosis. However, we did not perform a biopsy because the exact location of the tumour was not clear. Furthermore, since CCS tends to metastasize easily, we were concerned that a biopsy would lead to further metastasis. Although there was no proof that the tumour was CCS, if it was CCS, the treatment plan was based on a total resection. The decision on the treatment plan was difficult because the imaging findings were not fully clear to determine the boundaries of the lesion and he was asymptomatic. After much discussion by the heart team, we finally decided on a policy of surgical resection.

**Figure 3:**
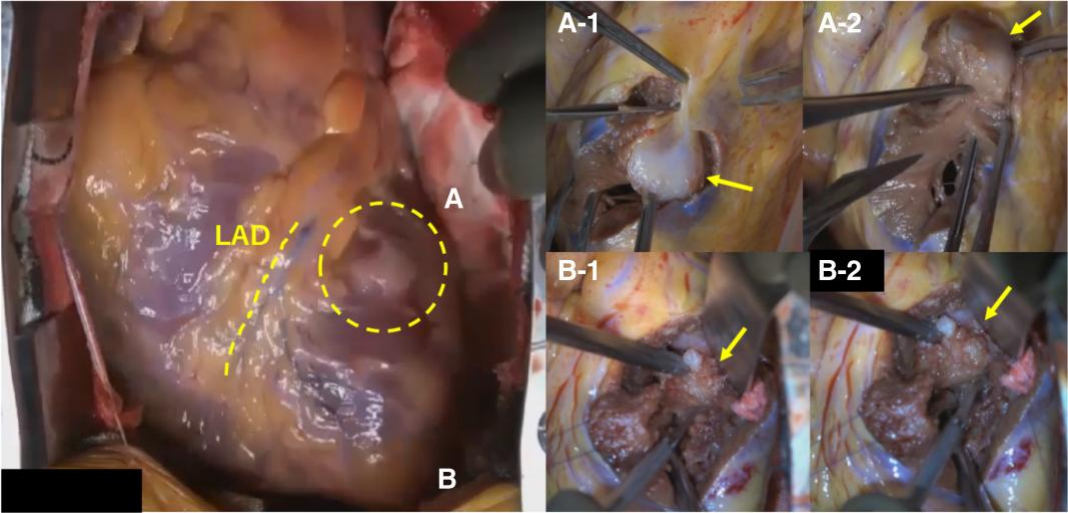
Surgical inspection. (*A*) On the surface of the heart, a whitish, well-defined lesion (circle) is seen in the middle of the anterolateral wall of the left ventricle, ∼1 cm to the left of the left anterior descending branch (LAD). The myocardium was incised along the left side of the LAD from the apex to the mass (arrow) in the middle of the anterolateral wall, and the tumour (arrow) was resected at the same site. (*B*) The mass between papillary muscles of left ventricular posterolateral wall is not exposed on the surface. After expanding the visual field and observing the intracardiac cavity, a white, well-defined tumour (arrows) was identified between the anterior and posterior papillary muscles.

**Figure 4:**
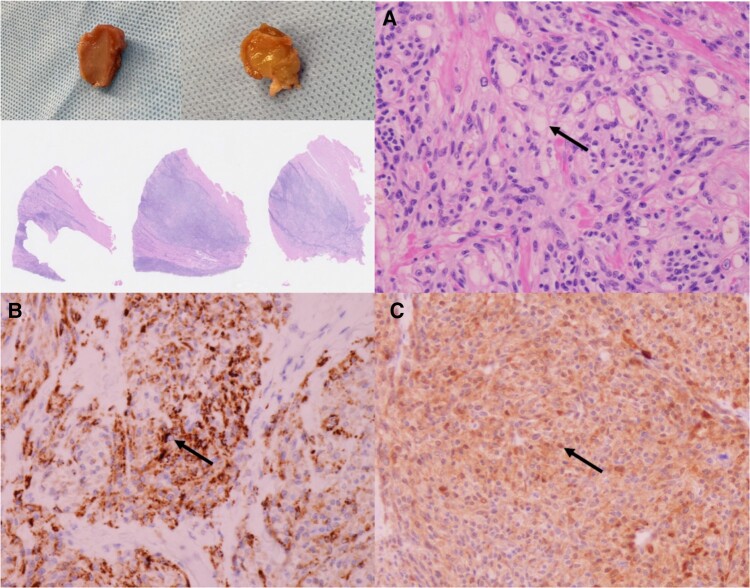
Pathological analysis. (*A*) Haematoxylin eosin stain shows clear nucleoli and some clear cytoplasm (arrow). (*B*) The mass is positive for HMB45 (human melanin black 45) (arrow). HMB45 is an antibody against the pre-melanosomal glycoprotein present in malignant melanoma and other tumours derived from melanocytes. (*C*) S100 is positive (arrow). It is an antibody against calcium-binding protein and is useful for staining nervous system tumours and malignant melanoma.

One month later, surgery was performed. After a median sternotomy, the anterior aspect of the heart was observed. A 15 mm large, white tone, well-defined lesion was found ∼1 cm to the left of the left anterior descending artery (LAD) in the middle of the anterolateral wall of the LV. On palpation, it was hard tissue and was considered to be one of the targeted neoplastic lesions. The tumour on the posterolateral wall was not exposed on the surface but was palpated under TOE-guided guidance and was also recognized as a firm neoplastic lesion. After determining that the tumour was resectable, the myocardium was incised along the left side of the LAD from the apex to the mass in the middle of the anterolateral wall, and the tumour was resected at the same site (*Figure 3A*). Although we were concerned that the boundary of the tumour might be unclear in the pre-operative evaluation, when we actually looked at it with the naked eye, the boundary was clear. We resected it with a margin and submitted it to rapid pathology. The mass in the middle of the anterolateral wall was identified as a sarcoma on intraoperative rapid pathology, and we were confident that it was a CCS. Therefore, we continued with resection and excision of the tumour on the endocardial surface between the papillary muscles of the posterolateral wall. After expanding the visual field and observing the intracardiac cavity, a white, well-defined tumour was identified between the anterior and posterior papillary muscles (*Figure 3B*). The tumour was enucleated using a scalpel after trimming the surrounding abnormal tendon cords, taking care not to damage the papillary muscles or tendon cords. The margins were measured during the procedure, and all were negative. The same confirmation was done for both masses. Pathological diagnosis revealed zonal or dense infiltrating and proliferating tumour tissue within the myocardial tissue of both lesions. The nucleolus was clear, with some light cytoplasm and no specific sequence, HMB45(+), S-100(+), and based on these findings, the diagnosis of a cardiac CCS was made (*Figure 4*). The post-operative course went well, and he was discharged 19 days later. He is still doing well, because the resection margins were negative, he did not receive any adjuvant therapy with radiation or chemotherapy. He is being followed up with PET-CT and TOE, and his cardiac function is good, with no recurrence or metastasis at the last follow-up 9 months after surgery.

## Discussion

Primary cardiac tumours are rare, <10% of which are malignant, sarcomas being the most common.^5^ Sarcomas account for 65% of malignant adult primary cardiac tumours, and their histopathological subtypes include angiosarcomas, leiomyosarcomas, and others.^6^ Metastatic cardiac tumours are 20–40 times more common than primary cardiac tumours, and melanomas have the greatest propensity for cardiac involvement.^7,8^ Clear cell sarcoma is a very rare disease, occurring primarily in the extremities, primarily in the lower extremities, primarily around the ankle, ankle, and knee joints, especially associated with tendons and periosteum.^9^ Although CCS is still a reasonably well-known disease in the orthopaedic field, its occurrence in the heart is rare and has rarely been reported in the past. The mainstay of treatment is extensive resection of the tumour. The beneficial effects of adjuvant chemotherapy and radiation therapy have not yet been fully evaluated.^10^ It is unclear whether the present lesion is a metastasis of a previous lesion or a primary lesion. We decided to resect because the lesion was confined to the heart and was not complicated by other organ involvement.

Clear cell sarcoma often metastasizes, and close examination and monitoring of lymph node, abdominal/pelvic, and skeletal metastases with CT, MRI, bone scan, and PET-CT are recommended. In this case, TTE, TOE, contrast-enhanced CT, PET-CT, MRI, and cardiac catheterization were performed. Transthoracic echocardiography is considered the first choice for imaging evaluation of cardiac involvement in patients with suspected metastases. In our case, however, the TTE findings were not specific and could not point to a mass. On MRI, CCS lesions typically seem hyperintense on T1-weighted images and may have heterogeneous signal on T2-weighted and late gadolinium enhancement images.^10,11^ In this case, delayed contrast was high signal, T1 mapping was different from normal myocardium, and mildly high signal was seen at T2-weighted, all of which is not consistent with previous literature. Cardiac MRI has been reported to show well-defined nodular lesions, but in this patient, the borders were indistinct. Positron emission tomography computed tomography images processed with glucose suppression allowed us to infer that there were two lesions, one in the middle of the anterolateral wall of the LV and the other between the papillary muscles of the posterolateral wall of the LV, and to compare them with MRI and TOE images. When acquired TOE images were retrospectively reviewed, the lesion area was hypoechoic compared to the normal myocardium. We consider it necessary to be fully aware of this fact and observe adequately with this in mind. The combination of multimodalities is highly effective in non-invasively revealing myocardial lesions and diagnosing CCS.

In summary, our patient had a CCS arising in the heart. Clear cell sarcoma is a rare disease, and even more rare to occur in the heart. This case was examined using multiple imaging modalities, including glucose-suppressed PET-CT and TOE, which ultimately assisted surgical resection and diagnosis of a CCS to treat the patient. It should be kept in mind that CCS can occur in the heart and be treated surgically by a personalized multi-modality imaging approach.

## Data Availability

Anonymous data will be shared upon request, and it is subjected to the data protection regulations.

## Lead author biography

Dr Ayano Osawa graduated from Hiroshima University, Japan. She currently works as a clinical staff in the Department of Cardiovascular Medicine at Hiroshima University Hospital, Hiroshima, Japan.
